# Incidence and Survival of Children and Adolescents With Wilms Tumor, United States, 2001–2020

**DOI:** 10.1002/cam4.70598

**Published:** 2025-02-10

**Authors:** Andres F. Espinoza, Ekene Onwuka, David A. Siegel, Shifan Dai, Sanjeev A. Vasudevan, Michael E. Scheurer, Philip J. Lupo

**Affiliations:** ^1^ Divisions of Pediatric Surgery and Surgical Research, Michael E. DeBakey Department of Surgery, Texas Children's Surgical Oncology Program and Liver Tumor Program, Dan L. Duncan Cancer Center Baylor College of Medicine Houston Texas USA; ^2^ Department of Pediatrics, Hematology‐Oncology Section, Texas Children's Hospital, Dan L. Duncan Cancer Center Baylor College of Medicine Houston Texas USA; ^3^ Division of Cancer Prevention and Control, National Center for Chronic Disease Prevention and Health Promotion Centers for Disease Control and Prevention Atlanta Georgia USA; ^4^ Cyberdata Technologies Inc. Herndon Virginia USA

## Abstract

**Background:**

Wilms tumor (WT) is the most common pediatric malignancy of the kidney. Past studies describing WT incidence and survival used surveillance data with < 30% of the US population. We evaluated differences in WT incidence and survival comparing demographic groups and tumor characteristics.

**Methods:**

We analyzed new cases of WT among patients aged < 20 years at diagnosis by using incidence data from US Cancer Statistics (USCS) for 2003–2020 and 5‐year relative survival (RS) data from the National Program of Cancer Registries (NPCR) for 2001–2019. To assess incidence trends, average annual percent change (AAPC) was calculated by using joinpoint regression. Relative survival (RS) and all‐cause survival were calculated overall and by demographic and clinical variables.

**Results:**

During 2003–2020, 8218 cases of WT were reported in USCS, which represented an age‐adjusted incidence rate of 5.7 cases per million. Rates were the highest among females (6.3), children aged 0–4 years (17.2), and non‐Hispanic Black patients (7.1). Overall, trends remained stable (AAPC = −0.4, 95% CI: −1.4 to 0.4). Among 7567 cases of WT in NPCR, 5‐year RS was 92.6%. Patients with the lowest survival include the following: those aged 10–19 years (hazard ratio [HR] = 1.65, 95% CI: 1.02–2.65); non‐Hispanic Black patients (HR = 1.39, 95% CI: 1.11–1.76); those with regional stage (HR = 1.93, 95% CI: 1.47–2.54) or distant stage (HR = 5.12, 95% CI: 3.99–6.57); and patients from nonmetropolitan counties (HR = 1.46, 95% CI: 1.09–1.96). Individuals diagnosed during 2011–2019 (HR = 0.64, 95% CI: 0.53–0.77) had higher survival than those diagnosed during 2001–2010.

**Conclusions:**

The highest WT incidence rates were patients who were female, 0–4 years, and non‐Hispanic Black. Survival improved during the study period; survival differed by race, ethnicity, metropolitan status, and age. Further studies to delineate the causes of these disparities may improve outcomes.

## 1 Introduction

Wilms tumor (WT) is the most common renal malignancy in children, most often presenting in those under age 5 years [1]. Since it was first described in 1899, epidemiological studies have investigated the incidence, risk factors, and outcomes associated with WT [1, 2, 3]. Established prognostic factors for relapse and death include anaplastic histology, loss of tumor heterozygosity on 1p and 11p15, and > 2 years of age at diagnosis [3]. These observations have been incorporated in risk stratification strategies, leading to notable gains in overall survival; however, survival was still < 90% for 25% of patients with WT [2, 3]. Studying epidemiological trends of WT provides understanding of risk factors for aggressive phenotypes and helps clinicians predict outcomes [1, 2, 3].

Past data from the Surveillance, Epidemiology, and End Results (SEER) database during 2002–2006 describe WT incidence as 5.4 cases per 1,000,000 children and adolescents [4]. Female sex and age under 5 years at time of diagnosis are two factors associated with higher incidence of WT [1, 2, 3]. In addition, studies focused on survival have largely been conducted using data from SEER database, which encompasses < 30% of the US population [5, 6, 7]. This, in turn, limits our understanding of current survival statistics, which are crucial for patient counseling and research efforts. New assessments are needed to guide novel risk stratification strategies. Therefore, we sought to evaluate the incidence of WT and survival of those diagnosed using recent population‐based surveillance data and to assess trends and disparities.

## 2 Methods

### 2.1 Data Source and Study Population

The US Cancer Statistics (USCS) database was used to assess the incidence of WT among patients aged 0–19 years at diagnosis from 2003 to 2020 in the United States. The USCS database captures all 50 states and the District of Columbia (DC), but Nevada and Indiana were excluded for not meeting data quality standards. Thus, the final USCS dataset encompassed 97% of the US population. North Dakota and Wisconsin were excluded from the race and ethnicity incidence analysis for not meeting data quality standards, and Kansas and Minnesota were excluded from the economic status because of state law. The National Program of Cancer Registries (NPCR) survival database was used to evaluate survival for patients diagnosed during 2001–2019. Five‐year survival was reported from the NPCR survival database. NPCR conducts active case follow‐up or linkage with the CDC's National Death Index and includes Alabama, Alaska, Arizona, Arkansas, California, Colorado, Delaware, Florida, Georgia, Idaho, Illinois, Kansas, Kentucky, Louisiana, Maryland, Maine, Minnesota, Mississippi, Missouri, Montana, Nebraska, New Hampshire, New Jersey, New York, North Carolina, North Dakota, Ohio, Oklahoma, Oregon, Pennsylvania, Rhode Island, South Carolina, Tennessee, Texas, Utah, Vermont, West Virginia, Wisconsin, and Wyoming and did not include Connecticut, Hawaii, Iowa, Indiana, Massachusetts, Michigan, New Mexico, Nevada, South Dakota, Virginia, Washington and the District of Columbia. The final dataset encompassed 83% of the US population [8]. Data from North Dakota and Wisconsin were excluded from the race and ethnicity relative survival analysis, and Kansas and Minnesota were excluded from the economic status relative survival analysis; all four states were excluded from the regression survival analysis. The International Classification of Childhood Cancer was used to define WT cases; cases included were limited to the “IV(a) nephroblastoma and other nonepithelial renal tumors” group and were additionally limited to *International Classification of Diseases for Oncology*, Third Edition (ICD‐O‐3) histology code 8960 [9]. Cases were limited to malignant cancers (behavior code = 3), microscopically confirmed cases, and only first primary sequence number.

### 2.2 Variables

We evaluated variables including sex, race and ethnicity, age at diagnosis, stage, and year of diagnosis. Race and ethnicity were grouped into non‐Hispanic (NH) White, NH Black, NH Asian or Pacific Islander (API), NH American Indian/Alaska Native (AI/AN), and Hispanic. Stage was defined as merged summary stage (local, regional, or distant) [10]; stage was defined independent of tumor laterality. In addition, we stratified by geography‐based variables, including county‐based socioeconomic status (SES), US census region, and metropolitan versus nonmetropolitan status. SES was grouped into five categories as defined by the Appalachian Regional Commission index‐based county economic classification system and was regrouped into three categories and described by percentile compared with other counties (top 25%, 25%–75%, and bottom 25%) [9]. Metropolitan versus nonmetropolitan status was defined by county size using Beale codes [11].

### 2.3 Statistical Analysis

Incidence rates (IRs) for each variable were expressed per 1 million persons and age‐adjusted to the 2000 US standard population. Incidence rate ratios (IRRs) were calculated for each variable. Temporal trends in incidence were described using average annual percent change (AAPC) calculated by joinpoint regression. A maximum of three joinpoints were used to determine a change in direction of the trend. Because the COVID‐19 pandemic caused data changes, the trend analysis of year 2020 was excluded [12].

In the survival analyses, outcomes were measured using 5‐year relative survival (RS), which aims to represent cancer survival in the absence of other causes of death. Relative survival is defined as the ratio of the observed survival of patients with cancer to the expected survival of a matched cohort of cancer‐free individuals and is calculated by using expected life tables that are stratified by age, sex, race and ethnicity, county‐level SES, geographic location (by US census region and metropolitan status), and calendar year of diagnosis. All‐cause survival curves, overall and by demographic and clinical variables, were generated using the Kaplan–Meier method [13]. Statistical testing for differences in survival curves was performed using the log‐rank test [13]. Multivariable Cox regression models were conducted to examine the effects of demographic and clinical variables on 5‐year all‐cause survival. Hazard ratios (HR) were generated for each variable, with a higher HR between compared groups indicating a higher risk of death.

The SEER*Stat 8.3.8 software program (National Cancer Institute, Bethesda, MD) was used to perform all analyses related to incidence and relative survival. All‐cause survival curves and related Cox regression models were generated using SAS Version 9.4 (SAS Institute Inc., Cary, NC). All tests were two‐sided and a *p* value ≤ 0.05 was considered statistically significant.

## 3 Results

### 3.1 Incidence

From 2003 to 2020, a total of 8218 new cases of WT were identified in the USCS database (Table 1). The incidence rate throughout this time was 5.7 per million (95% CI: 5.6–5.9). Female patients (6.3) had a higher incidence than males (5.2). The incidence during 2003–2020 was the highest among younger patients, in particular ages 1–4 years at 18.4 per million (95% CI: 17.9–18.9). NH Black patients were noted to have significantly higher IRR than NH White patients at 1.17 (95% CI: 1.11–1.24). By stage, incidence rates were the highest among the patients with localized disease compared to those with regional or distant stage (Table 1). As shown by IRR calculations, there were fewer differences in rates between metropolitan versus nonmetropolitan counties and also among the three county‐based economic status categories included in the study. Incidence was the highest in the Northeast and statistically and significantly higher than the West with an IRR of 0.88 (95% CI: 0.82–0.95) (Table 1).

**TABLE 1:** Incidence rate, incidence rate ratios, and average annual percent change in the United States Cancer Statistics database for Wilms tumor cases, age < 20 years, 2003–2020.

Variable	Count	Rate	95% CI	IRR	95% CI	*p*	AAPC	95% CI
Overall	8218	5.7	5.6–5.9				−0.4	−1.4 to 0.4
Sex								
Male	3785	5.2	5.0–5.3	Ref			−0.5	−1.9 to 1.1
Female	4433	6.3	6.2–6.5	1.23	1.17–1.28	< 0.05	0.1	−1.2 to 1.4
Age, years								
0–4	5991	17.2	16.8–17.7	Ref			−0.6^a^	−1.3 to 0
< 1	882	12.8	11.9–13.6	—			−0.8	−2.3 to 0.6
1–4	5109	18.4	17.9–18.9	—			‐0.6	−1.4 to 0.2
5–9	1856	5.3	5.1–5.5	0.31	0.29–0.32	< 0.05	1.0	−0.3 to 2.8
10–14	265	0.7	0.6–0.8	0.04	0.04–0.05	< 0.05	0.5	−2.3–5.7
15–19	106	0.3	0.2–0.3	0.02	0.01–0.02	< 0.05		
Race and ethnicity								
Non‐Hispanic White	4532	6.1	5.9–6.3	Ref			−1.0^a^	−1.8 to −0.2
Non‐Hispanic Black	1537	7.1	6.8–7.5	1.17	1.11–1.24	< 0.05	0.6	−0.8 to 2.1
Non‐Hispanic American Indian/Alaska Native	74	5.3	4.2–6.7	0.87	0.68–1.10	0.2674		
Non‐Hispanic Asian or Pacific Islander	225	2.8	2.5–3.2	0.46	0.40–0.53	< 0.05	−1.0	−3.6 to 1.9
Hispanic (all races)	1567	4.5	4.3–4.7	0.74	0.70–0.78	< 0.05	0.3	−1.0 to 1.7
Stage								
Localized only	3280	2.3	2.2–2.4	Ref			−0.6	−2.0 to 0.2
Regional	2694	1.9	1.8–2.0	0.82	0.78–0.87	< 0.05	−0.3	−2.9 to 2.3
Distant site(s)/node(s) involved	1978	1.4	1.3–1.4	0.61	0.57–0.64	< 0.05	1.0	−0.3 to 2.4
Metropolitan status								
Metropolitan counties	7034	5.8	5.6–5.9	—			−0.5	−2.2 to 1.6
Counties in metropolitan areas ≥ 1 million	4523	5.7	5.6–5.9	Ref			0.3	−0.4 to 1.1
Counties in metropolitan areas of 250,000 to 1 million	1786	5.8	5.5–6.1	1.01	0.96–1.07	0.7151	0.3	−1.3 to 2.1
Counties in metropolitan areas of < 250 thousand	725	5.8	5.4–6.2	1.01	0.94–1.10	0.7516	−1.0	−2.3 to 0.3
Nonmetropolitan counties	1128	5.7	5.4–6.1	1.00	0.93–1.06	0.9328	−0.1	−1.5 to 1.3
Economic status								
Top 25%	2599	5.7	5.5–5.9	Ref			0.3	−0.5 to 1.0
Middle 25%–75%	4495	5.8	5.6–6.0	1.01	0.97–1.06	0.5726	−0.2	−1.0 to 0.6
Bottom 25%	756	5.4	5.0–5.8	0.95	0.88–1.03	0.2273	−2.5	−5.6 to 0.4
US census region								
Northeast	1448	6.1	5.8–6.4	Ref			0.1	−1.8 to 2.0
Midwest	1663	5.8	5.6–6.1	0.96	0.90–1.04	0.3199	0.4	−1.3 to 2.1
South	3260	5.8	5.6–6.0	0.96	0.90–1.02	0.2020	−0.6	−2.0 to 0.9
West	1847	5.3	5.1–5.6	0.88	0.82–0.95	< 0.05	1.7	−0.5 to 3.5

*Note:* Variables with missing individual cases: Race and ethnicity (*n* = 169), Metropolitan status (*n* = 56), Stage (*n* = 36), US census region (*n* = 56).

Abbreviations: AAPC, average annual percent change; CI, confidence interval; IR, incidence rate; IRR, incidence rate ratios; Ref, reference.

^a^ Designates significant AAPC. AAPC calculation excludes year 2020.

Overall, the incidence was stable during 2003–2019 (AAPC −0.4, 95% CI: −1.4 to 0.4). NH White patients had a significant decrease in incidence (AAPC −1.0, 95% CI: −1.8 to −0.2), whereas incidence was stable for other race and ethnicity groups. Ages 0–4 years had a significant decrease in incidence (AAPC −0.6, 95% CI: −1.3 to 0), whereas incidence was stable for other age groups, including the subgroups of < 1 year and 1–4 years. In addition, there were no significant changes in incidence based on stage, metropolitan status, or county‐based economic status.

### 3.2 Survival

During the study period of 2001–2019, 7567 cases of WT were identified in the NPCR survival database (Table 2). The relative survival of our entire cohort was 92.6% (95% CI: 92.0–93.2). Although 95% confidence intervals overlapped, RS of male patients (93.4%, 95% CI: 92.4–94.2) was higher than female patients (92.0%, 95% CI: 91.0–92.8); RS was the highest among those aged < 1 year (95.7%, 95% CI: 93.8–97.0). Patients classified as NH Black had the lowest RS at 90.8% (95% CI: 89.0–92.3), whereas NH White patients had the highest RS at 93.1% (95% CI: 92.2–93.9). In addition, those with distant stage were noted to have the lowest RS at 84.0% (95% CI: 82.1–85.7). Confidence intervals overlapped among variables when stratifying RS by metropolitan status, county‐based economic status, and US census region.

**TABLE 2:** Five‐year survival for children and adolescents with Wilms tumor in the National Program of Cancer Registries database, 2001–2019.

Variable	Count	Relative survival (%)	95% CI	Hazard ratio (all‐cause survival)	95% CI	*p*
Overall	7567	92.6	92.0–93.2			
Sex						
Male	3471	93.4	92.4–94.2	Ref		
Female	4096	92.0	91.0–92.8	1.14	0.95–1.37	0.162
Age, years						
0–4	5569	93.7	93.0–94.3	—		
< 1	854	95.7	93.8–97.0	Ref		
1–4	4715	93.3	92.5–94.0	0.98	0.68–1.42	0.932
5–9	1660	90.2	88.5–91.6	1.15	0.77–1.70	0.496
10–14	240	86.6	81.1–90.5	1.65^a^	1.02–2.65	0.041
15–19	98	85.1	76.1–91.0			
Race and ethnicity						
Non‐Hispanic White	4258	93.1	92.2–93.9	Ref		
Non‐Hispanic Black	1436	90.8	89.0–92.3	1.39^a^	1.11–1.76	0.005
Non‐Hispanic American Indian/Alaska Native	70	93.0	83.2–97.2	1.16	0.70–1.94	0.562
Non‐Hispanic Asian or Pacific Islander	199	92.8	87.8–95.8			
Hispanic (all races)	1507	92.6	91.1–93.9	1.20	0.93–1.53	0.158
Stage						
Localized only	3026	96.8	96.0–97.4	Ref		
Regional	2429	93.9	92.8–94.8	1.93^a^	1.47–2.54	< 0.0001
Distant site(s)/node(s) involved	1801	84.0	82.1–85.7	5.12^a^	3.99–6.57	< 0.0001
Metropolitan status						
Metropolitan Counties	6507	92.9	92.2–93.5	—		
Counties in metropolitan areas ≥ 1 million	4237	93.2	92.3–93.9	Ref		
Counties in metropolitan areas of 250,000 to 1 million	1635	91.9	90.4–93.2	1.21	0.96–1.52	0.110
Counties in metropolitan areas of less than 250 thousand	635	93.1	90.6–94.9	1.11	0.77–1.60	0.578
Nonmetropolitan counties	1059	91.1	89.1–92.8	1.46^a^	1.09–1.96	0.012
Economic status						
Top 25%	2094	92.6	91.4–93.7	Ref		
Middle 25%–75%	4421	92.7	91.8–93.5	0.95	0.76–1.18	0.645
Bottom 25%	752	91.4	89.1–93.3	0.96	0.68–1.37	0.838
US census region						
Northeast	1360	93.6	92.1–94.9	Ref		
Midwest	1375	92.3	90.6–93.7	1.35	0.97–1.88	0.078
South	3001	92.5	91.4–93.4	1.17	0.88–1.54	0.277
West	1677	92.5	91.0–93.7	1.15	0.85–1.56	0.356
Year of diagnosis						
2001–2010	3927	91.6	90.7–92.4	Ref		
2011–2019	3640	94.0	93.0–94.8	0.64^a^	0.53–0.77	< 0.0001

*Note:* Variables with missing individual cases (*n*): Race and ethnicity (*n* = 97), Stage (*n* = 311).

Abbreviations: CI, confidence interval; Ref, reference.

^a^ Designates significant hazard ratio.

Regression analysis was performed using all‐cause survival. Patients aged 10–19 years had a higher likelihood of death compared to those aged < 1 year (HR = 1.65, 95% CI: 1.02–2.65). NH Black patients had a lower all‐cause survival (90.5%) than NH White (92.9%) (HR = 1.39, 95% CI: 1.11–1.76). Patients with regional disease (93.5%) (HR = 1.93, 95% CI: 1.47–2.54) and distant disease (83.7%) (HR = 5.12, 95% CI: 3.99–6.57) had lower all‐cause survival than with localized disease (96.6%). Patients from nonmetropolitan counties had lower all‐cause survival (90.6%) than from metropolitan areas of ≥ 1 million (92.9%) (HR = 1.46, 95% CI: 1.09–1.96). Individuals diagnosed during 2011–2019 (93.8%) (HR = 0.64, 95% CI: 0.53–0.77) had higher all‐cause survival than those diagnosed during 2001–2010 (91.3%). There were no significant differences in all‐cause survival based on sex or US Census region.

**FIGURE 1:**
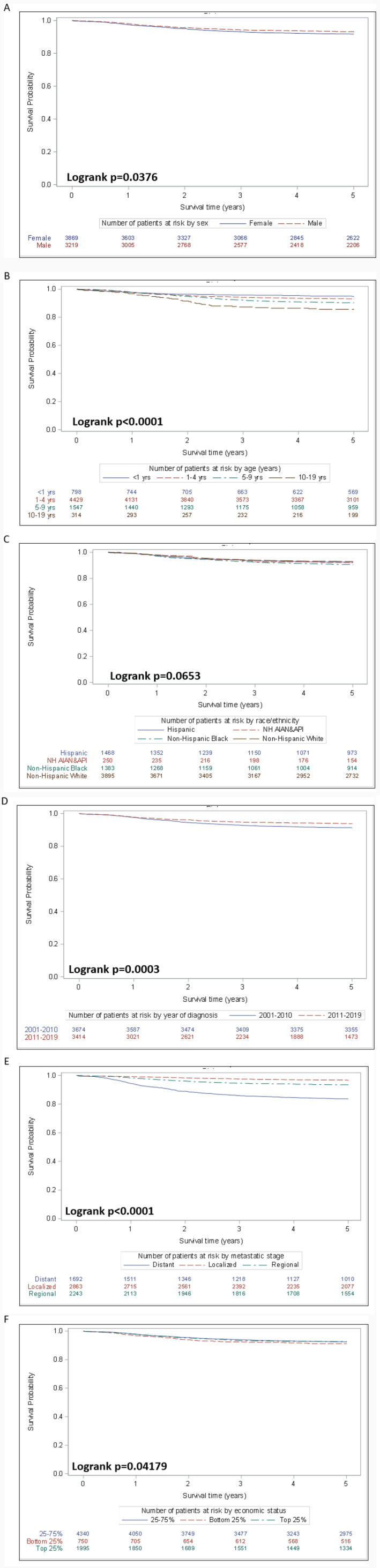
Kaplan–Meier survival estimation curve for children and adolescents with Wilms tumor by (A) sex, (B) age, (C) race and ethnicity, (D) year of diagnosis, (E) stage, and (F) economic status.

When considering all‐cause survival and using Kaplan–Meier curves, there was lower all‐cause survival among females and patients diagnosed and treated before 2011, and significant differences in curves based on age, stage, and county‐based economic status (Figure 1). All‐cause survival was not significantly different among patients of different race and ethnicity groups, and metropolitan status (Figure 1; Figure S1).

## 4 Discussion

In our population‐based assessment, we demonstrated that the overall incidence of WT has been stable with a higher incidence noted in younger patients, NH Black patients, those of female sex, and those with localized disease. We also found that RS was > 92%. Patients that were more likely to have lower survival included those of older age, specifically those aged 10–19 years, NH Black patients, and those from nonmetropolitan counties.

Previous studies have indicated that the incidence of several pediatric solid tumors is increasing [1, 2, 3, 7, 14]; however, this was not the case for WT in our data. Our incidence data is consistent with previous studies of WT that showed an overall higher incidence among female and younger patients [7, 14, 15]. However, the slightly higher overall incidence and decrease in incidence among patients aged 0–4 years in our study compared to Horner and colleagues may be from differences in population coverage between SEER and USCS (< 30% compared to 97%), and differences in years included [4]. We also provide additional support to the previously reported higher incidence of WT among females and those who are NH Black [1, 2, 3, 7, 14, 15, 16]. The reason for the higher incidence in these groups is likely multifactorial, and both genetic and environmental factors are proposed contributors [17, 18, 19]. For example, given that some tumors have been found to have mutations on the X chromosome, it is possible that an epigenetic modification on the X chromosome that has not been identified can lead to the development of WT [20]. Another hypothesis is that mutations of the tumor suppressor gene, *WT1*, leads to development of WT [21, 22, 23]. Mutations of *WT1* increases estrogen levels, which predisposes renal parenchyma to developing WT [21, 22, 23]. Further efforts to clearly determine drivers among this high‐risk population are ongoing, and results should be informative when creating future screening and treatment algorithms [24].

Our data show that most pediatric patients were diagnosed with localized WT. It is possible that improved screening and increases in less aggressive histology may be driving this pattern [1, 25]. Improved screening guidelines for genetically predisposing conditions guide care for patients with these conditions; recommended monitoring via imaging and tumor markers help clinicians identify WT early given the higher risk of developing WT among these patient populations, increasing the detection of localized disease [1, 25].

The survival of the patients in our cohort continued to improve, which is consistent with findings from the National Wilms Tumor Study (NWTS) group [26, 27, 28, 29]. The NWTS has continuously implemented several new therapies and scientific discoveries to create novel treatment strategies and improve risk stratification [30]. Nonetheless, our study showed that patients with distant disease and older age persistently exhibit 5‐year survival percentages that approximate 84% and 85%, respectively. It is possible that tumors in this patient population may have anaplastic biology or aggressive mutations that lead to chemo‐resistance [29, 30, 31, 32]. Several studies have found similar findings in other pediatric solid tumors [33, 34]. A focused effort to elucidate pathways that may render these tumors resistant should inform targeted or combination therapies for treatment‐refractory cancers.

Disparities have been a focus for several years concerning outcomes among both adult and pediatric populations. It is well‐documented that disparities in survival exist in WT among NH B0lack patients and those living in nonmetropolitan counties [35, 36]. Studies that have attempted to identify the cause of these disparities report that lack of close access to providers, lack of insurance, and inability to obtain follow‐up care have been proposed as the drivers [35, 36]. Lack of access to providers and insurance may limit referrals in the health system which decreases access to clinical trials [35, 36, 37]. Similarly, the inability to obtain follow‐up care may limit close monitoring and treating relapsed disease [37]. While it is likely that a combination of factors is associated with living in a nonmetropolitan county; these studies and continued surveillance might be helpful in guiding future interventions to address disparities [35, 36].

Our study has limitations that should be taken into consideration. Given that this is a cancer registry‐based investigation, clinical and biological information were not available to be included in our analysis. Thus, we could not consider factors known to affect outcomes, such as the presence of anaplastic histology or loss of tumor heterozygosity on 1p and 11p15, which are known poor prognostic factors. In addition, chemotherapeutic adherence, appropriate surgical control, and trial enrollment results have been shown to improve survival, but these variables are not available in these databases. For this manuscript, we were limited to using staging definitions available in USCS and NPCR (e.g., merged summary stage, which defined disease as localized, regional, and distant disease), as opposed to staging systems used in clinical oncology (e.g., the Children's Oncology Group system, which defines stage I through stage V). Finally, as noted in the methods, some states were excluded from components of the USCS incidence and NPCR survival databases for not meeting data quality standards (i.e., North Dakota, Wisconsin) or because of state law (i.e., Kansas, Minnesota). Despite these limitations, the results presented in our manuscript support previous findings and provide additional trends that may aid in improving care for patients with WT.

In conclusion, this study used two separate national cancer registry databases to evaluate the frequency and survival for children diagnosed with WT. We present that WT appears to have lower survival in those of older age. These data can guide work in at‐risk populations being conducted by providers, hospitals, clinical trial organizations, researchers, and public health professionals. We provide further data to demonstrate that disparities continue among NH Black patients and those of nonmetropolitan counties. These results can help clinicians and clinical researchers understand how to develop new interventions to help improve quality of life and prolong survival. Public health initiatives to address these disparities may include outreach efforts for at‐risk individuals of disparities in outcomes and to encourage clinical trial enrollment. More actions may include efforts to increase access to long‐term care services at the national or statewide level, which can provide access to patients experiencing comorbid medical conditions as a result of their treatment.

## Author Contributions

**Andres F. Espinoza:** conceptualization (equal), data curation (equal), formal analysis (equal), investigation (equal), methodology (equal), validation (equal), writing – original draft (equal), writing – review and editing (equal). **Ekene Onwuka:** conceptualization (equal), formal analysis (equal), funding acquisition (equal), investigation (equal), methodology (equal), validation (equal), visualization (equal), writing – original draft (equal). **David A. Siegel:** data curation (equal), formal analysis (equal), investigation (equal), supervision (equal), validation (equal), writing – original draft (equal). **Shifan Dai:** data curation (equal), formal analysis (equal), validation (equal), writing – original draft (equal). **Sanjeev A. Vasudevan:** investigation (equal), visualization (equal), writing – original draft (equal). **Michael E. Scheurer:** investigation (equal), methodology (equal), visualization (equal), writing – original draft (equal). **Philip J. Lupo:** conceptualization (equal), formal analysis (equal), investigation (equal), methodology (equal), visualization (equal), writing – original draft (equal), writing – review and editing (equal).

## Disclosure

The findings and conclusions in this report are those of the authors and do not necessarily represent the official position of the Centers for Disease Control and Prevention.

## Ethics Statement

This activity was determined by CDC not to be human subjects research requiring IRB approval.

## Conflicts of Interest

The authors declare no conflicts of interest.

## Supporting information


**Figure S1.** Kaplan–Meier survival estimation curve for children and adolescents with Wilms tumor by metropolitan status.

## Data Availability

The data that support the findings of this study are available on request by contacting uscsdata@cdc.gov.
