# Supplementary figures and images for: Incidence and Survival of Children and Adolescents With Wilms Tumor, United States, 2001–2020
Source: Cancer Med. 2025 Feb 10;14(3):e70598. doi: 10.1002/cam4.70598 (PMC11809554; doi:10.1002/cam4.70598)

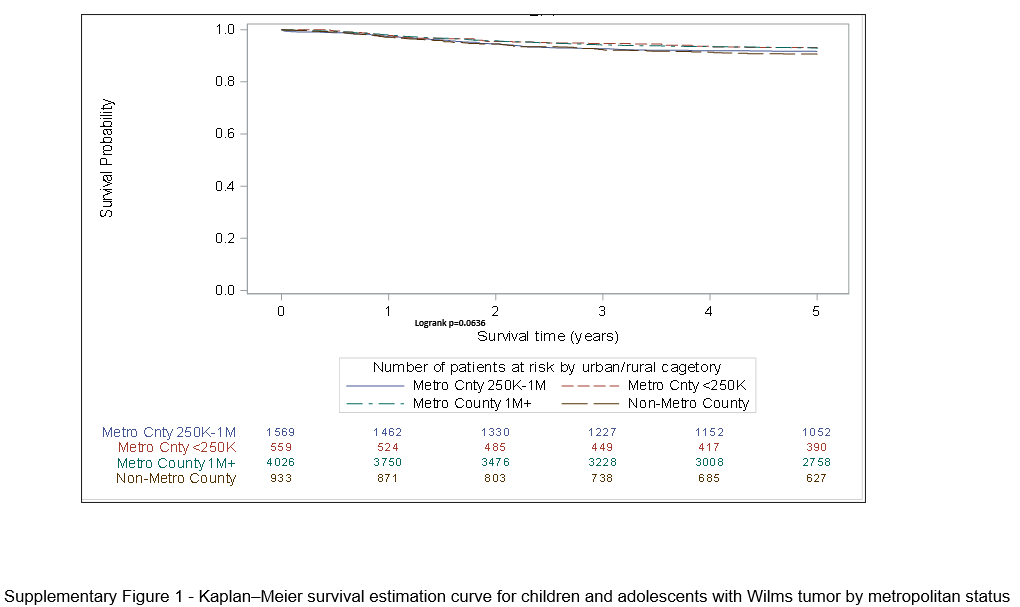

Supplement: Supplementary file 1 — Figure S1. Kaplan–Meier survival estimation curve for children and adolescents with Wilms tumor by metropolitan status. [file CAM4-14-e70598-s001.docx]
